# Free-Wilson in the 21st Century – evolution of a versatile toolkit for SAR analysis

**DOI:** 10.1186/1758-2946-3-S1-P6

**Published:** 2011-04-19

**Authors:** Brad Sherborne

**Affiliations:** 1 MSD, Newhouse, Scotland, ML5 1SH, UK

## 

When drug discovery projects are transferred, then rapidly assessing the available SAR in both overview and in numerical detail is a prerequisite for effective computational chemistry input. Following multiple company acquisitions, regular requirements for the approach has led to an effective and fast platform for Free-Wilson [1] based SAR review: in fact the utility is sufficient that it can for a useful reporting or project browsing tool for the busy modeller. Figure 1

**Figure 1:**
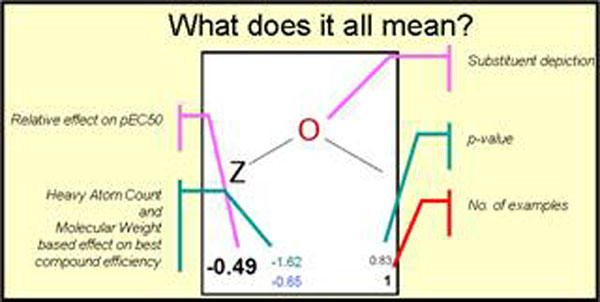


In this talk it is shown how a carefully crafted PDF report can be combined with “on the fly” Matched Molecular Pair [2] and other drill downs to provide a versatile platform for SAR exploration and presentation that is appealing and to medicinal chemists.

## References

[B1] Free SM Jr Wilson JW A Mathematical Contribution to Structure-Activity Studies J Med Chem 1964 7 395 399 10.1021/jm00334a001 14221113

[B2] Leach AG Jones HD Cosgrove DA Kenny PW Ruston L MacFaul P Wood JM Colclough N Law B Matched molecular pairs as a guide in the optimization of pharmaceutical properties; a study of aqueous solubility, plasma protein binding and oral exposure J Med Chem 2006 49 6672 6682 10.1021/jm0605233 17154498

